# The effects of recreational use of marijuana in adolescent brain health: A review

**DOI:** 10.1192/j.eurpsy.2021.368

**Published:** 2021-08-13

**Authors:** G. Kurnijuanto, T. Kantohe

**Affiliations:** Faculty Of Medicine, Sam Ratulangi University, Manado, Indonesia

**Keywords:** Marijuana, adolescent, Brain

## Abstract

**Introduction:**

Marijuana is widely used among people, recreationally and medically. However, recent studies have shown that Marijuana has negative effects on brain structures and functions.

**Objectives:**

To discuss the effects of Marijuana use on brain development in adolescence.

**Methods:**

The method that is used in this study is literature review, through analyzing and summarizing the data that were collected from PubMed, epidemiology articles from BNN and CDC, and other online journals to understand the effects of Marijuana on the brain development in adolescence. There were 25499 articles that were filtered and screened resulting in 10 articles that were used as data of this literature review.

**Results:**

Marijuana effects on the brain are divided into structural changes and functional changes. Structural changes are seen in the brain hemispheres, amygdala, hippocampus, and nucleus accumbens. While functional changes are seen in behavioral and cognitive changes in everyday life and even psychotic disorders.

**Conclusions:**

Marijuana use has shown negative effects on the human body, organs that are rich in cannabinoid receptors, especially the Brain. Therefore, Marijuana use among adolescents may disrupt their developing brain, and cause adolescents to have structural and functional changes in the brain.

**Disclosure:**

No significant relationships.

